# Clinical Development of c-MET Inhibition in Hepatocellular Carcinoma

**DOI:** 10.3390/diseases3040306

**Published:** 2015-10-28

**Authors:** Joycelyn J. X. Lee, Jack J. Chan, Su Pin Choo

**Affiliations:** Division of Medical Oncology, National Cancer Centre Singapore, 11 Hospital Drive, Singapore 169610, Singapore; E-Mails: joycelyn.lee.j.x@nccs.com.sg (J.J.X.L.); jack.chan.j.j@nccs.com.sg (J.J.C.)

**Keywords:** hepatocellular carcinoma (HCC), c-MET, hepatocyte growth factor (HGF), c-MET inhibitor, tivantinib, cabozantinib

## Abstract

Hepatocellular carcinoma (HCC) is one of the leading causes of cancer death. In patients with advanced or unresectable HCC, there are few treatment options. Conventional chemotherapy has limited benefits. Sorafenib, a multi-kinase inhibitor, improves survival, but options for patients intolerant of or progressing on sorafenib are limited. There has been much interest in recent years in molecular therapeutic targets and drug development for HCC. One of the more promising molecular targets in HCC is the cellular-mesenchymal-epithelial transition (c-MET) factor receptor. Encouraging phase II data on two c-MET inhibitors, tivantinib and cabozantinib, has led to phase III trials. This review describes the c-MET/hepatocyte growth factor (HGF) signalling pathway and its relevance to HCC, and discusses the preclinical and clinical trial data for inhibitors of this pathway in HCC.

## 1. Introduction

Hepatocellular carcinoma (HCC) is the second leading cause of cancer-related death worldwide [1]. Treatment options in advanced HCC are limited, with conventional chemotherapy having limited efficacy. The multi-kinase inhibitor, sorafenib, was established as the standard of care in patients with advanced HCC after two randomised trials showed a significant survival benefit [2,3], but its use is generally limited to those with good hepatic reserves, and five-year survival remains dismal at less than 10%. Outside of clinical trials, there is a dearth of approved therapeutic options for patients who have disease progression on sorafenib.

At least three recent phase III trials of molecularly targeted agents as second-line treatment in HCC failed to meet their primary endpoints. These trials studied brivanib (BRISK-PS) [4], everolimus (EVOLVE-1) [5], and ramucirumab (REACH) [6] compared to placebo.

The tyrosine kinase receptor, cellular-mesenchymal-epithelial transition (c-MET) factor receptor, has been studied as a potential therapeutic target, and phase II data with the c-MET inhibitors, tivantinib and cabozantinib, have been encouraging, prompting ongoing phase III trials.

In this review, we discuss the c-MET/HGF pathway, its relevance to HCC, and summarise the preclinical and clinical data to date regarding c-MET inhibitors in HCC.

## 2. c-MET Pathway and Relevance in HCC

### 2.1. The HGF/c-MET Pathway

The MET proto-oncogene was first identified in an osteosarcoma cell line [7]. The gene encodes for a transmembrane receptor tyrosine kinase (RTK), also known as c-MET, for which HGF is a ligand [8].

Binding of HGF to c-MET’s Sema domain leads to receptor homodimerisation, autophosphorylation of tyrosine residues in the tyrosine kinase domain, and downstream activation of the Ras/MAPK, PI3K/Akt, and Ras/Rac/Pho pathways [9]. These promote cell proliferation, survival, migration, and angiogenesis [10].

### 2.2. Abnormalities in HGF/c-MET Signalling Pathways in Cancer

Abnormal activation of HGF/c-MET signalling can occur in several ways [11,12]:

(1) Overexpression of HGF

Elevation of HGF protein levels, both intratumoural and systemic, has been noted in many tumour types, such as lung cancer (50%), breast cancer (91%), stomach cancer (87%), colon cancer (95%), cancer of the head and neck (45%) and liver cancer (33%) [13]. Elevated plasma levels of HGF have been suggested to correlate with a poor prognosis for several forms of cancer, including HCC [14].

Plasma HGF levels have been consistently shown to be higher in patients with HCC compared to controls. Biomarker analyses using samples from the SHARP trial [2] and phase II trial for tivantinib [15,16] suggest that HGF levels may have prognostic significance, with better survival in patients with lower levels, and decreasing levels suggesting disease response.

(2) Overexpression of c-MET

Overexpression of c-MET in tumour tissue has been noted in many cancers, such as lung cancer, stomach cancer, breast cancer, kidney cancer, colon cancer, and HCC.

In HCC, high c-MET expression has also been found to be a poor prognostic marker, correlating with poorly differentiated tumours and lower survival rates [15,16]. Tumour c-MET expression was also predictive of response to tivantinib.

(3) MET amplification

High MET gene copy number can be due to general ploidy status, or true focal gene amplification. MET amplification is less common than overexpression of the protein receptor tyrosine kinase [17], but has been noted primarily in gastrointestinal cancers such as gastric cancer, oesophageal cancer [18] and colon cancer, as well as in endometrial carcinoma, medulloblastoma, non-small cell lung cancer (NSCLC) [19] and gliomas [20].

(4) Activating mutations

Activating mutations in c-MET’s tyrosine kinase domain have been reported in hereditary and sporadic papillary renal cell carcinomas [21], paediatric liver cancer and squamous cell carcinoma of the head and neck. Other mutations within c-MET’s juxtamembrane region or its Sema domain, where HGF binds, have also been noted in gastric cancer, breast cancer, pleural mesothelioma, and small-cell lung cancer. In addition, c-MET also interacts with other key oncogenic signalling pathways [22].

The interaction between c-MET and HER2 family members is well-documented. MET amplification has been reported to lead to EGFR tyrosine kinase inhibitor (TKI) resistance by HER3-mediated activation of PI3K/AKT signalling in NSCLC [23,24]. MET amplification has also been reported as a mechanism for resistance for colorectal cancer patients treated with anti-EGFR antibodies [25,26].

c-MET/HGF signalling promotes angiogenesis through increasing vascular endothelial growth factor (VEGF)-A expression and interaction with the VEGF receptor (VEGFR) pathway [27]. It has also been shown to maintain the stem cell niche in cancer, with WNT activity in colorectal cancer stem cells described to be supported by myofibroblast-secreted HGF [28].

## 3. Overview of HGF/c-MET Pathway Inhibitors

Small molecule c-MET inhibitors can be classified as selective inhibitors, which specifically target c-MET tyrosine kinase in an ATP-competitive or non-competitive manner, or non-selective inhibitors, which target other kinases in addition to c-MET.

**Figure 1:**
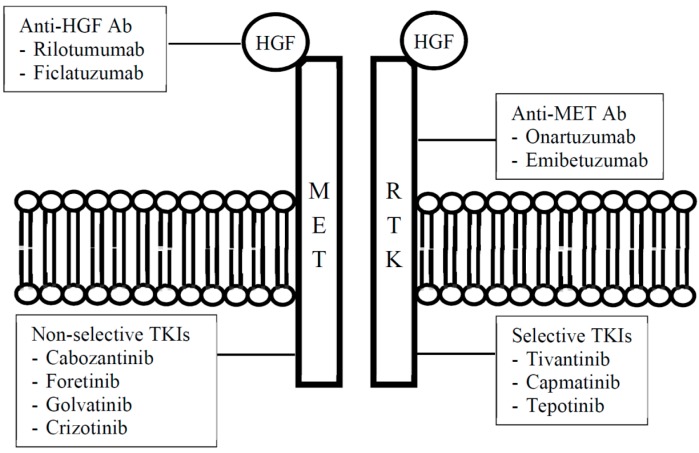
Classification of HGF/c-MET inhibitors.

Alternatively, blockade of the HGF/c-MET pathway can also be effected through anti-HGF neutralising antibodies, which block only HGF-dependent c-MET activation, or anti-MET antibodies (Table 1, Figure 1). Anti-HGF antibodies will not be discussed further in this paper owing to a lack of reported signal of activity in HCC.

**Table 1:** Classification of HGF/c-MET Inhibitors.

Type of Inhibitor	Drug	
	Name	Synonym(s)
**Antibody**		
Anti-HGF	Rilotumumab	AMG 102
	Ficlatuzumab	AV-299
Anti-c-MET	Ornartuzumab	MetMAb
	Emibetuzumab	LY2875358
**Small molecule inhibitor**		
Selective	Tivantinib	ARQ 197
	Capmatinib	INC280 (formerly INCB028060)
	Tepotinib	MSC2156119J, EMD 1214063
Non-selective	Cabozantinib	XL184
	Foretinib	GSK1363089 (formerly XL880)
	Golvatinib	E7050
	Crizotinib	PF-2341066

## 4. Preclinical Studies of c-MET Inhibitors in HCC

Studies have shown the role of HGF in enhancing liver regeneration, hepatocyte survival and tissue remodelling after acute injury [29], and in suppressing hepatocyte apoptosis [28].

In human HCC cell lines, c-MET positive cells were noted to have cancer stem cell-like characteristics. Treated with c-MET inhibition however, c-MET positive cells had increased apoptosis, decreased proliferation and suppressed tumour growth, while c-MET reduced cells survived the inhibition treatment. This suggests that c-MET inhibition may be an effective therapy only for selected patients with strong c-MET expression [30].

c-MET pathway activation is also postulated to promote cancer metastasis by inducing epithelial-to-mesenchymal transition (EMT) [31], which causes epithelial cells to lose E-cadherin and cell-to-cell contact and acquire mesenchymal characteristics such as motility and invasion. HGF treatment has been found in murine models of HCC to induce EMT and sustain a mesenchymal phenotype [32].

## 5. Clinical Studies of c-MET Inhibitors in HCC

c-MET inhibitors are sequenced in this section by how extensively they have been investigated in HCC, in descending order. Selected active clinical trials involving these agents are summarised in Table 2.

**Table 2:** Selected Active Clinical Trials on c-MET inhibitors for HCC.

Drugs	Phase	Patient Selection	Trial Status	ClinicalTrials.gov Identifier
*Tivantinib (ARQ 197)*				
Monotherapy	I	Advanced solid tumours ^†^	Recruiting	NCT02150733
With bevacizumab	I	Advanced solid tumours	Active, not recruiting	NCT01749384
With temsirolimus	I	Advanced solid tumours	Recruiting	NCT01625156
With topotecan	I	Advanced solid tumours	Active, not recruiting	NCT01654965
Tivantinib *vs.* Placebo	III	MET-high HCC	Recruiting	NCT02029157
Tivantinib *vs.* Placebo	III	MET-high HCC	Recruiting	NCT01755767
*Cabozantinib (XL 184)*				
Cabozantinib *vs.* Placebo	III	HCC	Recruiting	NCT01908426
*Capmatinib (INC280)*				
Monotherapy	I	Advanced solid tumours	Recruiting	NCT01546428
Monotherapy	I	MET-dysregulated solid tumours	Recruiting	NCT01324479
Monotherapy	II	MET-dysregulated HCC (1st line)	Recruiting	NCT01737827
*Tepotinib (MSC2156119J*, *EMD 1214063)*				
Monotherapy	I	Advanced solid tumours	Active, not recruiting	NCT01014936
Monotherapy	Ib/II	MET + HCC (1st line) ^‡^	Recruiting	NCT01988493
Monotherapy	Ib/II	MET + HCC	Recruiting	NCT02115373
*Golvatinib (E7050)*				
With sorafenib	Ib/II	HCC (1st line) ^§^	Active, not recruiting	NCT01271504
*Onartuzumab (MetMAb)*				
Monotherapy	I	Advanced solid tumours	Recruiting	NCT02031731
*Emibetuzumab (LY2875358)*				
Monotherapy	I	Advanced solid tumours	Active, not recruiting	NCT01287546
With Ramucirumab	Ib/II	Advanced solid tumours	Recruiting	NCT02082210

All trials are for second-line therapy of advanced HCC unless otherwise indicated. ^†^ Restricted to patients with hepatic impairment; ^‡^ Randomised against sorafenib; ^§^ Phase II portion of study randomised against sorafenib alone.

### 5.1. Tivantinib (ARQ 197)

Tivantinib is an oral non-ATP-competitive selective small molecular inhibitor of c-MET. Binding of tivantinib to c-MET stabilises the receptor in its inactive conformation, hence blocking both ligand-dependent and ligand-independent receptor phosphorylation and thus reducing downstream signalling [33,34]. Tivantinib demonstrated anti-tumour activity in a wide range of tumour cell lines, as well as in xenograft models [34].

#### 5.1.1. Phase I Studies

Phase I studies for tivantinib, both as monotherapy [35,36,37] and as combination therapy with gemcitabine [38], erlotinib [39] and sorafenib [40], have been conducted in advanced solid tumours. The mechanism of action of tivantinib has been questioned preclinically but Yap *et al.* showed that c-MET decreases in tumours treated with tivantinib [36].

In the phase Ib trial by Santoro *et al.* [41], tivantinib monotherapy was studied in previously treated HCC patients with Child-Pugh A or B liver cirrhosis. Notably, liver function did not worsen in these patients. Aside from a higher rate of neutropaenia (any grade 52%, grade 3/4 38%), tivantinib demonstrated a manageable safety profile. Out of 21 patients, none achieved any objective response, though nine achieved the best response of stable disease.

The combination of tivantinib and sorafenib was examined in a phase I study of 20 HCC patients by Martell *et al.* [40], following the report of synergistic anti-proliferative activity with the combination in preclinical studies [42]. Of note, eight patients in the study had received sorafenib and/or sunitinib previously, and five achieved stable disease or better. The aforementioned trials suggest that tivantinib may be a promising second-line treatment for advanced HCC.

Chai *et al.* performed a pooled analysis to summarise the outcomes of 53 patients with HCC or biliary tract cancer receiving tivantinib in phase I trials [43]. These included 23 patients receiving tivantinib monotherapy and 30 patients receiving tivantinib in combination with a second drug. The overall response rate (ORR) and disease control rate (DCR) were 6% and 62%, respectively.

#### 5.1.2. Phase II Study

Based on the phase I data, tivantinib was studied as a second-line therapy for advanced HCC compared against placebo in a randomised multi-centre phase II trial in 107 patients with Child-Pugh A cirrhosis [15]. All of the patients had previously received sorafenib, except for four who had received sunitinib, and had progressed on or did not tolerate first-line treatment. Crossover to open-label tivantinib was allowed for patients on placebo at radiological progression. Of note, the initial dose of tivantinib was planned for 360 mg twice daily, but this was decreased to 240 mg twice daily after 57 patients were enrolled because of a high incidence of grade 3 and 4 neutropaenia. In the whole population, the trial met its primary end point as tivantinib improved time to tumour progression (TTP) (1.6 month *vs.* 1.4 month; hazard ratio, HR 0.64). Progression-free survival (PFS) and overall survival (OS) however were not statistically different.

Pre-specified subgroup analysis according to c-MET expression status was performed, with MET-high defined as more than 50% of HCC cells with 2+ or stronger staining intensity on immunohistochemistry (IHC) [15]. Only the subgroup of patients with MET-high tumours showed a significant survival benefit with improvement in median TTP (2.7 months *vs.* 1.4 month; HR 0.43; 95% CI 0.18–0.81), median PFS (2.2 months *vs.* 1.4 month; HR 0.45; 95% CI 0.21–0.95) and median OS (7.2 months *vs.* 3.8 months; HR 0.38; 95% CI 0.18–0.81). The survival advantage for patients on the lower dose of 240 mg twice daily of tivantinib was at least equivalent to that observed among patients on the higher dose of 360 mg twice daily. Among MET-low patients, however, there was no difference in response rates or survival outcomes between tivantinib and placebo, suggesting that c-MET expression is predictive of response to tivantinib.

The same authors subsequently showed that the interaction test between treatment and tumour c-MET levels in OS was positive (*p* = 0.0385) [16]. They also found that tumour c-MET was the only biomarker which predicted response to tivantinib.

Selected phase I and II studies of tivantinib monotherapy are summarised in Table 3.

**Table 3:** Selected phase I and II clinical trials of Tivantinib monotherapy in HCC (adapted from Rimassa *et al.* [44]).

Trial	Study Design	Patient Selection	Toxicity Outcomes	Efficacy Outcomes	Dose
*Phase I studies*					
Rosen *et al.*,(2011) [35]	Dose-escalation study	Advanced solid tumours (*N* = 79)	Most common AE: fatigue (14%), nausea (14%), vomiting (10%), anaemia (8%), diarrhoea (6%)	Three patients (3.8%) achieved PR; 40 patients (50.6%) maintained SD for a median of 19.9 weeks	MTD not reached R2PD: 360 mg BD
			DLT: leucopaenia, neutropaenia, thrombocytopaenia, vomiting, dehydration in 2 patients treated with 360 mg BD		
Yap *et al.*, (2011) [36]	Dose-escalation study	Advanced solid tumours (*N* = 51)	Most common AE (>10%): grade 1/2 fatigue (16%), nausea (14%), vomiting (12%)	Best response of SD ≥ 4 months in 14 patients (27%)	MTD/R2PD: 360 mg BD
Santoro *et al.*, (2013) [41]	Phase Ib study	HCC (*N* = 21), including Child-Pugh A (*N* = 17) or B (*N* = 4) liver cirrhosis	No drug-related worsening of liver function	Best response of SD in nine patients (43%)	RP2D: 360 mg BD
			Grade ≥ 3 drug-related AEs in 11 patients (52%), including neutropaenia in eight patients (38%)		
			Grade 5 neutropaenic septic shock (*N* = 1)		
			Four cardiac events were considered possibly or probably related to study drug		
*Phase II study*					
Santoro *et al.*, (2013) [15]	Placebo-controlled randomised phase II study; crossover allowed at radiologic PD (*N* = 23)	Advanced HCC (*N* = 107) failing or intolerant of first-line systemic therapy with sorafenib or sunitinib	Most common AE: asthenia (42%), loss of appetite (27%), neutropaenia (21%), fatigue (12%)	Increased TTP for the ITT population (6.9 *vs.* 6.0 weeks). Greatest clinical benefit for MET-high patients: TTP (11.7 *vs.* 6.1 weeks), PFS and OS (7.2 *vs.* 3.8 months)	240 mg BD

Abbreviations: PD: progressive disease; AE: adverse events; DLT: dose-limiting toxicities; PR: partial response; SD: stable disease; TTP: time to progression; ITT: intention-to-treat; PFS: progression free survival; OS: overall survival; MTD: maximum tolerated dose; RP2D: recommended phase 2 dose; BD: twice daily.

#### 5.1.3. Phase III Studies

Extending from the phase II data, there are currently two phase III double-blind, randomised controlled trials that are recruiting patients with advanced HCC and high c-MET-expression to compare tivantinib as second-line treatment against placebo, namely the METIV-HCC trial in the West (ClinicalTrials.gov Identifier: NCT01755767) and the JET-HCC trial in Japan (ClinicalTrials.gov Identifier: NCT02029157).

In the METIV-HCC trial, the original dose of 240 mg twice daily was reduced to 120 mg twice daily, after a higher than expected rate of neutropaenia and higher than expected drug exposure levels were observed. These were attributed to the switch in formulation from capsules used in the phase II study to tablets [45,46]. The primary endpoint of the trial is OS, with secondary endpoints defined as PFS and safety.

### 5.2. Cabozantinib (XL 184)

Cabozantinib is a non-selective oral multi-kinase inhibitor targeting c-MET, VEGFR2, KIT, RET, FLT3 and TIE-2. Cabozantinib has been shown to prolong survival in a c-MET-driven transgenic mouse model of HCC and to show efficacy against human HCC xenografts grown in mice [47].

#### 5.2.1. Phase I Study

A phase I dose-escalation study of cabozantinib in 85 patients with advanced solid tumours established the maximum tolerated dose (MTD) at 175 mg daily [48]. Dose-limiting toxicities (DLT) were hand-foot syndrome, mucositis, and transaminitis. The study included one patient with HCC whose disease was measurable, and in whom cabozantinib attained stable disease for at least three months.

#### 5.2.2. Phase II Study

A phase II randomised discontinuation study evaluated cabozantinib in advanced solid tumours of nine different tumour types, including HCC (*N* = 41) [49,50]. The study design incorporated a 12-week “lead-in” treatment period with cabozantinib followed by open-label continued treatment in responders until disease progression, treatment discontinuation in patients with disease progression, and random blinded assignment between cabozantinib and placebo in those with stable disease. The most frequent grade 3 and higher adverse events associated with cabozantinib were hand-foot syndrome (15%), diarrhoea (9%), and thrombocytopaenia (9%). DCR at 12 weeks was 71% in patients with HCC [50]. Notably, 49% of these patients were sorafenib-naïve, that is, cabozantinib was the first-line therapy for these patients.

Of note, this study did not evaluate for c-MET expression as a predictor of response to cabozantinib, and given the broad spectrum of targets of cabozantinib, it is unclear how much of the activity is attributable to c-MET inhibition alone. In fact, the combined inhibitory effects of c-MET and VEGF may be particularly effective, which is postulated to be due to upregulated c-MET signalling from VEGF inhibition, either from resultant hypoxia or direct interactions between VEGFR2 and MET [51].

Selected phase I and II studies of cabozantinib monotherapy are summarised in Table 4.

**Table 4:** Selected phase I and II clinical trials of Cabozantinib monotherapy in HCC.

Trial	Study Design	Patient Selection	Toxicity Outcomes	Efficacy Outcomes	Dose
*Phase I study*					
Kuzrock *et al.*, (2011) [48]	Dose escalation study	Advanced solid tumours (*N* = 85)	DLT: HFS, mucositis, transaminitis	In one patient with HCC whose disease was measurable, SD for at least three months	MTD: 175 mg OD
*Phase II study*					
Cohn *et al.*, (2012) [50]	Randomised discontinuation study	HCC (*N* = 41)	Most common grade ≥ 3 AE: HFS (15%), diarrhoea (9%), thrombocytopaenia (9%)	DCR at 12 weeks: 71% (Asian subgroup: 77%)	100 mg OD

Abbreviations: DLT: dose-limiting toxicities; HFS: hand foot syndrome; AE: adverse events; SD: stable disease; DCR: disease control rate; MTD: maximum tolerated dose; OD: once daily.

#### 5.2.3. Phase III Study

Given the encouraging data from the phase II study, a phase III randomised double-blind study is currently recruiting to compare cabozantinib against placebo as second-line treatment for advanced HCC patients who have previously received sorafenib [52]. Enrolment started in September 2013 with a target recruitment of 760 patients. (ClinicalTrials.gov Identifier: NCT01908426). Endpoints of the study are OS (primary), PFS and ORR (secondary), with two interim analyses and a final analysis planned.

### 5.3. Capmatinib (INC280, Formerly INCB028060)

Capmatinib is a highly selective c-MET inhibitor. It has demonstrated strong dose-dependent anti-tumour activity and dose-dependent reduction of phosphorylated MET (pMET) levels in c-MET-dependent murine tumour models [53].

#### 5.3.1. Phase I Study

In a phase I dose-escalation study, capmatinib was tested in 33 patients with confirmed c-MET-dysregulated advanced solid tumours, with HCC representing the commonest tumour type (45%), which were refractory to current therapy or for which effective therapy was lacking [54]. Stable disease was reported in 8/33 (24%) of the entire cohort. The recommended phase II dose (RP2D) was 600 mg twice a day, with DLT of fatigue and hyperbilirubinaemia.

#### 5.3.2. Phase II Studies

A phase II trial is currently ongoing testing the efficacy and safety of capmatinib as first-line treatment for patients with c-MET-dysregulated advanced HCC (ClinicalTrials.gov Identifier: NCT01737827). The trial is actively recruiting patients.

There was also a phase II randomised trial for capmatinib as second-line treatment for patients with advanced HCC after sorafenib, but the trial was suspended without any patient recruitment (ClinicalTrials.gov Identifier: NCT01964235).

### 5.4. Tepotinib (MSC2156119J, EMD 1214063)

Tepotinib is a specific, reversible, ATP-competitive c-MET inhibitor.

#### 5.4.1. Phase I Study

Following encouraging preclinical data in liver cancer models [55], a phase I study of tepotinib demonstrated good anti-tumour activity and tolerability in patients with advanced solid tumours [56]. DLT were asymptomatic lipase and amylase increases, nausea and vomiting, fatigue, and ALT elevation. The RP2D was 500 mg per day.

#### 5.4.2. Phase II Studies

There are currently two phase Ib/II trials ongoing for tepotinib. The first is a single-arm trial evaluating tepotinib as second-line treatment for MET-positive advanced HCC (ClinicalTrials.gov Identifier: NCT02115375). The other trial is a randomised open-label trial comparing tepotinib against sorafenib as upfront treatment in Asian patients with MET-positive advanced HCC (ClinicalTrials.gov Identifier: NCT01988493). MET positivity in the trial is defined as moderate or strong protein overexpression on IHC [57].

### 5.5. Foretinib (GSK1363089, Formerly XL880)

Foretinib is an ATP-competitive TKI with activity against c-MET, AXL, RON, VEGFR2, TIE-2 and PDGFR. It has been shown to inhibit tumour growth and prolong mouse survival in patient-derived HCC xenograft models [58].

#### 5.5.1. Phase I Study

A phase I study of foretinib showed DLT of transaminitis and elevated lipase levels, and common adverse events of hypertension, fatigue, diarrhoea and vomiting, proteinuria, and haematuria [59]. The RP2D was 250 mg given on the first five days of a 14-day cycle.

#### 5.5.2. Phase I/II Study

Foretinib has been studied as first-line therapy in a phase I/II study in Asian patients with advanced HCC. The phase I portion of the study showed ORR of 24%, disease stabilisation rate of 79% and median time to progression of 4.2 months, with no DLT observed at 30 mg once daily [60].

### 5.6. Golvatinib (E7050)

Golvatinib is a non-specific c-MET inhibitor, with activity also against VEGFR2, c-KIT and RON. It has been shown to promote tumour regression and prolong survival in mouse xenograft models [61].

#### 5.6.1. Phase I Studies

Two dose-finding phase I studies were conducted in patients with advanced solid tumours. The Japanese study found that the MTD was 200 mg twice a day [62], while the UK study determined the MTD as 400 mg once daily [63]. The DLT in both studies were similar, being derangements in liver enzymes, fatigue, and nausea and vomiting, with the former study also reporting proteinuria in 50% of the study cohort.

#### 5.6.2. Phase Ib/II Study

A phase Ib/II clinical trial is currently recruiting, in which the phase II cohort will compare golvatinib plus sorafenib against sorafenib alone as first-line use in patients with advanced HCC (ClinicalTrials.gov Identifier: NCT01271504). The phase I portion of the trial suggested that the golvatinib and sorafenib combination had manageable toxicity and showed an encouraging 17% of patients with partial responses and durable stable disease in another 31% [64].

### 5.7. Onartuzumab (MetMAb)

Onartuzumab is a monovalent, humanised monoclonal antibody specific for an epitope in the HGF-binding domain of the c-MET receptor. It was developed to overcome the limitation of bivalent antibodies which was thought might cause receptor dimerisation [65]. Onartuzumab forms a stable bond with c-MET on the cellular surface without inducing c-MET internalisation or shedding [66].

#### Phase I Studies

A phase I dose-escalation study of onartuzumab as a single agent and in combination with bevacizumab was carried out in patients with advanced solid malignancies. The maximum tolerated dose was not reached, while the most common drug-related adverse events included fatigue, peripheral oedema, nausea and hypoalbuminaemia [67]. A second phase I study of onartuzumab in Japanese patients with solid tumours showed no DLT when used alone or in combination with erlotinib [68]. A third phase I study of onartuzumab specific to Chinese patients with advanced or metastatic solid tumour is currently recruiting patients (ClinicalTrials.gov Identifier: NCT02031731).

A phase Ib open-label study evaluating onartuzumab as a single agent and in combination with sorafenib in patients with advanced HCC has completed recruitment (ClinicalTrials.gov Identifier: NCT01897038).

### 5.8. Emibetuzumab (LY2875358)

Emibetuzumab is a bivalent c-MET-specific monoclonal antibody that blocks HGF binding to c-MET, and neutralises and accelerates internalisation and degradation of the c-MET receptor upon binding, decreasing its level of cell surface expression [69].

#### 5.8.1. Phase I Study

A phase I dose-escalation study of emibetuzumab was performed in advanced solid tumours, establishing a RP2D of 750 mg every 2 weeks [70]. No DLT were observed, with the most frequent adverse effects reported being nausea, vomiting and diarrhoea.

#### 5.8.2. Phase Ib/2 Study

A phase Ib/2 study examining emibetuzumab in combination with ramucirumab in advanced solid tumours including HCC is actively recruiting (ClinicalTrials.gov Identifier: NCT01602289).

## 6. Biomarkers for c-MET-Targeted Therapies

It is unclear whether systemic levels of HGF predict response to anti-HGF/c-MET therapies. HGF levels are also known to be elevated in many other clinical settings including infections, graft-*versus*-host disease, and after surgical procedures.

Use of IHC for determination of c-MET protein overexpression has been extensively reviewed [71,72], but the variability among published studies in current literature suggests that standardisation of protocols is warranted [12]. Identifying MET-positive/high patients using IHC has been studied as part of various trials, such as the phase II trials of ornatuzumab or placebo with erlotinib in advanced NSCLC [73], rilotumumab or placebo combined with chemotherapy in advanced gastric or gastric oesophageal cancer [74], and tivantinib or placebo in advanced HCC [15,16]. The three trials defined different IHC cut-off criteria: at least 50% tumour cells with 2+ or 3+ staining was referred to as “MET diagnostic positive” in the ornatuzumab study and “MET-high” in the tivantinib study, whereas the rilotumumab study defined “MET-positive” as at least 25% membrane staining of tumour cells at any intensity.

Determination of pMET as a biomarker has been used *in vitro*, but has not been validated in larger studied in histological specimens.

In the onartuzumab trial [73], additional subgroup analyses were performed to determine the effect of MET copy number changes and EGFR mutational status. c-MET IHC was found to correlate with MET FISH, but benefit to c-MET-targeted therapy was seen in patients positive by IHC but negative by FISH. In the same study, pMET expression was also studied, but many cases with moderate to strong signals for c-MET by IHC were negative for pMET, suggesting that pMET is an insufficiently sensitive biomarker. In both studies, high c-MET expression was found to be prognostic, and interestingly, low c-MET expression seemed to be predictive of poorer outcome when treated with targeted therapy, emphasising the need for good assays and biomarkers to select a population suitable for targeted therapies.

Another possible strategy is the assessment of MET sequence status, including MET mutations, MET amplification, and chromosome 7 polysome [75].

Preclinical studies of different c-MET inhibitors revealed variable efficacy based on the specific MET mutation, e.g., PF-2341066/4217903, has greater activity against certain c-MET ATP-binding site mutations compared to c-MET kinase domain activations [76]. One such TKI, SU11274, has also showed selective inhibition for two of four identified MET mutations [77]. MET amplification (defined as MET:CEP7 ratio ≥ 2) has been found to correlate with increased clinical response of metastatic gastric cancer to foretinib in a phase II study [78]; whereas MET copy number (positives scored as ≥ four copies in ≥ 40% of cells), to correlate with increased clinical response to tivantinib with erlotinib in advanced NSCLC [79].

## 7. Conclusions

Therapeutic options for patients with advanced HCC intolerant to or progressive on sorafenib are scant. Data from c-MET inhibitors is promising, with phase III trials in progress for tivantinib and cabozantinib.

Considering that preclinical and clinical data suggest that the benefit of c-MET inhibition may be restricted to a patient subpopulation with high c-MET expression, future trials may need to be enriched by prospectively incorporating biomarker analyses to validate this hypothesis, so as to better select patients who would benefit from these therapies. Already, the METIV-HCC trial has adopted such a strategy of enrolling only patients with MET-high HCC, and its results are eagerly awaited.
